# Pharmacologic management of renal involvement in monogenic autoinflammatory diseases

**DOI:** 10.1186/s40001-026-03936-6

**Published:** 2026-02-12

**Authors:** Ahmed Fayed, Mohamed Tharwat Hegazy, Jurgen Sota, Carla Gaggiano, Gaafar Ragab

**Affiliations:** 1 Nephrology Unit, Internal Medicine Department, Cairo University, Cairo, Egypt; 2 Rheumatology and Clinical Immunology Unit, Internal Medicine Department, Cairo University, Cairo, Egypt; 3 Department of Medical Sciences, Surgery and Neurosciences, Research Center of Systemic Autoinflammatory Diseases and Behçet’s Disease Clinic, University of Siena, Siena, Italy

**Keywords:** Monogenic, Autoinflammatory disorders, Kidney, Drugs, Nephrotoxicity, Inflammasome, Amyloidosis

## Abstract

Kidney involvement represents one of the main targets of the systemic inflammatory process and is underscored by a heterogeneous pathology ranging from amyloidosis to non-amyloid-related damage rooted in inflammasome activation. A thorough approach to monogenic autoinflammatory disorders (AIDs) includes identifying phenotypic patterns, comprehending pathophysiology, and customizing therapy to improve patients' quality of life. In this review, we will discuss some pharmacological aspects focusing on the kidney as a target in AIDs. A special emphasis will be placed on medications used for AIDs cases with renal impairment, as well as drug-induced nephrotoxicity in monogenic AIDs. Understanding the mechanisms of drug-induced nephrotoxicity and applying preventive measures are critical for safely managing monogenic AIDs with renal involvement.

## Introduction

In 1999, Dr. Daniel Kastner first introduced the concept of autoinflammatory disorders (AIDs). AIDs are characterized by seemingly unprovoked attacks of systemic inflammation in the absence of antigen-specific T cells or autoantibodies. These attacks typically present with fever and systemic symptoms such as rash, arthritis, serositis, lymphadenopathy, hepatosplenomegaly, central nervous system, as well as renal involvement. Innate immunity plays the primary role in the pathogenesis of AIDs in contrast to the adaptive immunity which is a key factor in autoimmune illnesses [1]. Monogenic AIDs refer to conditions that originate from genetic mutations acting on a single gene important in managing inflammation. Since its first description, more than 40 genes connected to AIDs have been found; these genes have an impact on several innate immune system components [2, 3]. The most common and canonic monogenic AIDs comprise familial Mediterranean fever (FMF), tumor necrosis factor receptor–associated periodic fever syndrome (TRAPS), hyperimmunoglobulin D syndrome/mevalonate kinase deficiency (MKD), and cryopyrin-associated periodic syndromes (CAPS). The review aimed to highlight the mechanisms and implications of drug-induced nephrotoxicity in patients with AIDs, emphasizing the need for careful management and preventive measures.

## Review methodology

**Fig. 1:**
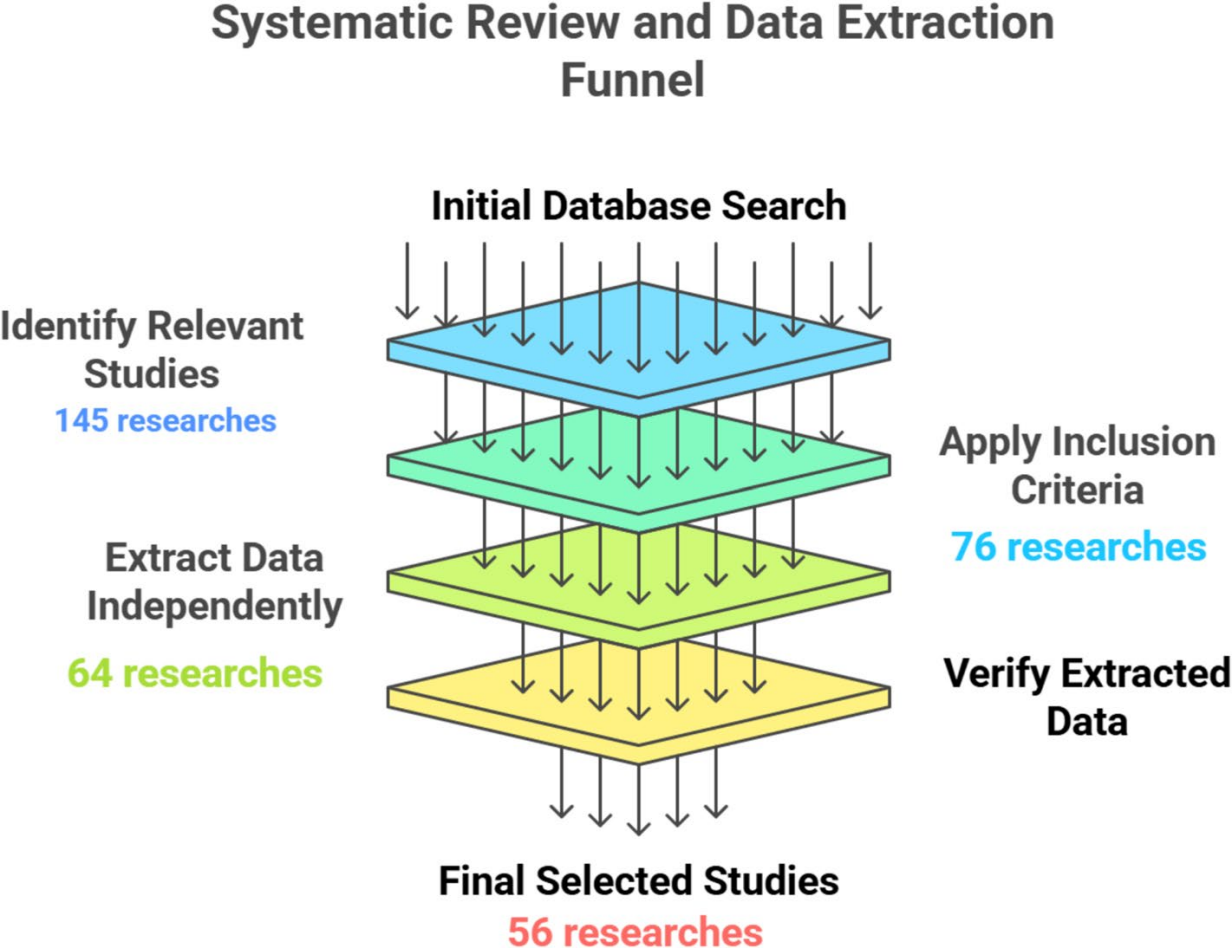
Illustrated systematic review and data extraction funnel

This research was carried out in the form of a systematic review using the PRISMA guidelines on reporting systematic reviews and meta-analysis. They are presented in a PRISMA flow diagram (Fig. 1).

Data sources and search strategy:

We did an extensive search of six electronic databases, PubMed/MEDLINE, Embase, LILACS, Scopus, Web of Science, and The Cochrane Library. A search was done in grey literature with Google Scholar and ProQuest dissertations and theses, as well as a manual search of reference lists. The most recent date on which the search strategy has been updated was June 2024. There were no restrictions on language, date of publication, or follow up period.

Eligibility criteria

Studies were included if they met the following criteria:

1. Dedicated to monogenic autoinflammatory diseases with respect to drug-induced nephrotoxicity.
2. Has included human subjects, of any age.
3. Reported first observations of studies (e.g., cohort, cross-sectional or case–control surveys) or clinical trials.
4. Reported renal outcome (e.g. proteinuria, hematuria, decreased glomerular filtration rate, histologic finding).

Exclusion criteria were:

1. Commentaries, editorials or opinion pieces.
2. Research that does not mention kidney involvement.
3. Research on renal diseases unassociated with autoinflammatory conditions.
4. Non-human studies or studies in vitro.

When a given publication represented more than one case–control population, then each population was identified and analyzed independently.

Study Selection and Data Extraction:

Two reviewers were used to screen titles and abstracts. Potentially eligible studies were selected using full texts. Conflicts were managed as an agreement or through a third reviewer. Two authors extracted the data without any interaction and the third author verified the accuracy and reliability of the data.

Risk of bias and evidence grading:

The risk of bias in studies that pasted prevalence, or observational data were evaluated by Joanna Briggs Institute (JBI) Critical Appraisal Checklist desiring the study design. The overall certainty of the evidence in the individual studies assessing each of the outcomes of interest was determined using the Grading of Recommendations, Assessment, Development, and Evaluation (GRADE) approach.

The quality of the included studies regarding methodology was tested using the JBI checklist where it was found that there are very few studies with low methodological quality and most of the studies remained moderate and high-quality with certain issues raised in regard to sample size and biases. The external evidence on the effectiveness of biologic therapy in monogenic autoinflammatory diseases involving the kidney was produced as moderate based on international criteria GRADE, because of a variation in the design of the studies and the limited sizes of samples.

## The kidney as a target in monogenic autoinflammatory diseases (AIDs)

**Fig. 2:**
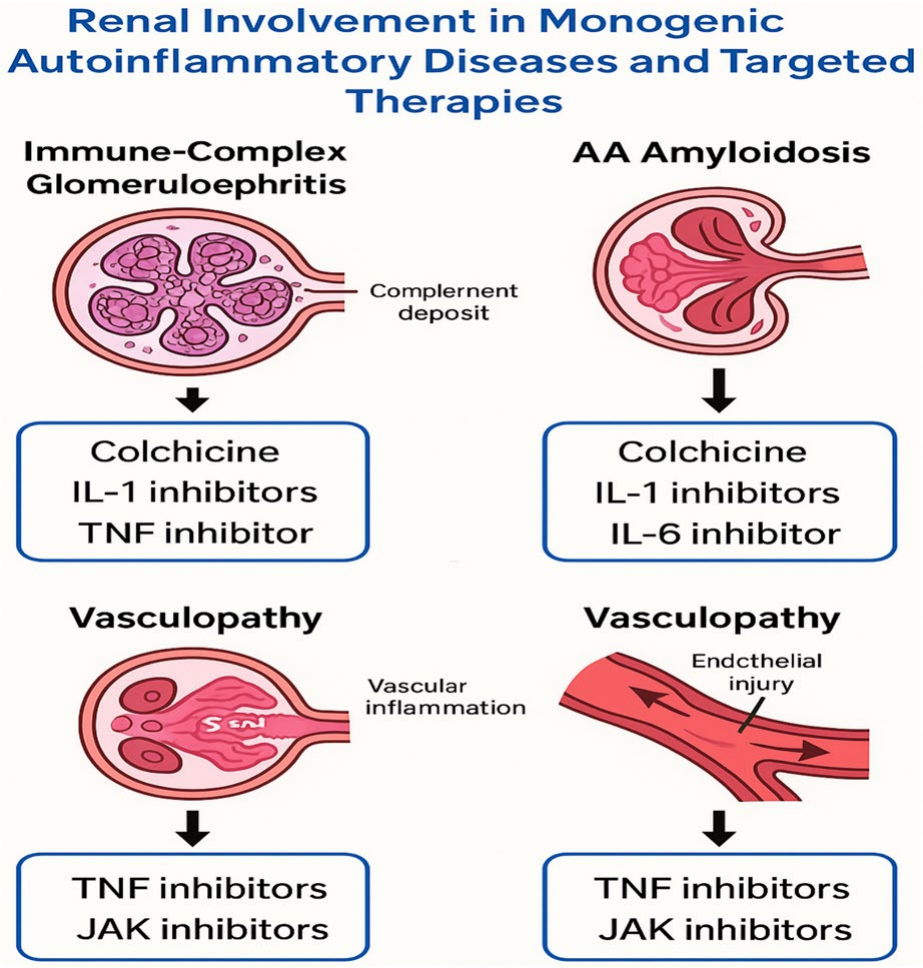
Renal involvement in Monogenic Autoinflammatory Diseases and targeted therapies. Renal involvement in monogenic autoinflammatory diseases (AIDs) encompasses a spectrum of pathological patterns, ranging from AA amyloidosis to non-amyloid-associated glomerular, tubular, and vascular injury. Cytokine overproduction of IL-1,6 and TNF-α leads to glomerular and endothelial injury. Colchicine, IL-1, Il-6, TNF, JAK inhibitors are important lines for treatment. IL: Interleukin, TNF: tumor necrosis factor, JAK: Janus kinase

“Renal involvement in monogenic autoinflammatory diseases (AIDs) encompasses a spectrum of pathological patterns, ranging from AA amyloidosis to non-amyloid-associated glomerular, tubular, and vascular injury (Fig. 2) [4]. While inflammasome activation [5] (e.g., NLRP3, NLRC4, Pyrin) contributes significantly through downstream IL-1β release [6], the underlying pathophysiology is more complex and multifactorial. Additional mechanisms include:

- **Cytokine overproduction** (e.g., IL-6, TNF-α, IFN-α/β) leads to glomerular and endothelial injury [7]**.**
- **Defective apoptotic pathways**, as seen in TRAPS (TNFRSF1A mutations), resulting in prolonged inflammatory cell survival and oxidative stress [8]**.**
- **Endothelial dysfunction and vasculitis**, particularly in ADA2 deficiency (DADA2) and interferonopathies like STING-Associated Vasculopathy (SAVI) [9]**.**
- **Hyperresponsiveness of innate immune receptors**, causing a sustained inflammatory milieu and immune complex deposition (e.g., IgA nephropathy in FMF) [4]**.**
- **Cytokine storms and macrophage activation syndrome (MAS)**, contributing to acute tubular necrosis or thrombotic microangiopathy in MAS-like settings (e.g., NLRC4-MAS, Deficiency of IL-1 Receptor Antagonist (DIRA) [4]**.**

These pathways interact synergistically to drive kidney damage, and their dominance may vary depending on genetic mutation, disease subtype, and patient age” [2–6] (Table 1).

**Table 1:** Monogenic autoinflammatory diseases (AIDs) with renal involvement- clinical, genetic, and pathophysiologic features

Disease	Clinical renal features	Associated gene(s)	Mechanistic pathway
FMF (Familial Mediterranean Fever) [4, 23, 24]	AKD, NAKD (FSGS, Mesangial proliferative GN, IgA nephropathy, crescentic GN, diffuse proliferative GN, PAN-like presentation (renal vasculitis)) [20, 21]	*MEFV*	Inflammasome activation (Pyrin/NLRP3), IL-1β overproduction
MKD (Mevalonate Kinase Deficiency/HIDS) [4]	AKD	*MVK*	Dysregulated isoprenoid pathway → impaired apoptosis, IL-1β excess
TRAPS (TNF Receptor–Associated Periodic Syndrome) [4, 5]	AKD, segmental GN, proteinuria	*TNFRSF1A*	Defective TNF receptor shedding → NF-κB overactivation, mitochondrial ROS
CAPS (Cryopyrin-Associated Periodic Syndromes) [5]	AKD, proteinuria	*NLRP3*	Constitutive NLRP3 inflammasome activation → IL-1β surge
DADA2 (Deficiency of Adenosine Deaminase 2) [9]	Polyarteritis-like renal vasculitis, infarcts	*ADA2* (also *CECR1*)	Endothelial dysfunction, TNF-driven vasculitis, macrophage skewing
SAVI (STING-Associated Vasculopathy) [9]	Proteinuria, renal vasculopathy (rare)	*TMEM173* (STING)	Type I interferonopathy → JAK-STAT activation, endothelial injury
NLRC4-MAS	GN (rare), AKI during MAS episodes	*NLRC4*	Inflammasome-mediated IL-18 overproduction, cytokine storm
DIRA (Deficiency of IL-1 Receptor Antagonist) [4]	Proteinuria, AKI (reported)	*IL1RN*	Uncontrolled IL-1α/β signaling due to loss of receptor antagonist

*FMF* Familial Mediterranean fever, *AKD* amyloid kidney disease, *NAKD* nonamyloid kidney diseases, *FSGS* Focal segmental glomerulosclerosis, *GN* glomerulonephritis, *PAN* polyarteritis nodosa, *MKD* mevalonate kinase deficiency, *CAPS* cryopyrin-associated periodic syndromes, *TRAPS* TNF receptor-associated periodic fever syndrome, *DADA2* Deficiency of Adenosine Deaminase 2, *SAVI* STING-Associated Vasculopathy, *DIRA* Deficiency of IL-1 Receptor Antagonist

### Pharmacotherapy and management challenges in monogenic AIDs with renal involvement

Renal phenotypes in monogenic AIDs comport the renal involvement that involves amyloidosis, immune complex glomerulonephritis, vasculitis, as well as tubulointerstitial nephritis. The treatment of these patients should have a two-pronged approach in treating dynamic inflammation in the body and renal sequelae.

• Treatment Overview

Targeted anti-inflammatory treatment is the mainstay of treatment and must be based on disease subtype, genetics and renal profile:

- Colchicine is the drug of choice in the treatment of Familial Mediterranean Fever (FMF) being very helpful to prevent crises and control subclinical inflammation. It also decreases the serum amyloid A (SAA) and thus hinders or reverses early amyloidosis [10, 11].
- IL-1 inhibitors (e.g. anakinra, canakinumab) play an important role in colchicine-resistant FMF, TRAPS, CAPS and MKD. These drugs inhibit the IL-18 pathway of inflammasome activation and they are especially useful in renal amyloidosis patients [10–15] (Table 3).
- TNF-alpha inhibitors (e.g. etanercept, adalimumab, infliximab) have been reported to be effective in AIDs with vasculopathy (e.g. DADA2), and those with glomerular or vascular renal damage [10].
- Tocilizumab as an IL-6 inhibitor has shown response to SAA mediated renal disease and systemic AIDs characterized by elevated IL-6 [16].
- JAK inhibitors (e.g., tofacitinib, baricitinib) have been promising in type I interferonopathies (such as SAVI and CANDLE) and may manifest/exhibit renal vasculopathy but **cannot** be used in severe CKD as they are renally excreted [17] (Table 3).

• Challenges and Unmet Needs

Despite therapeutic advancements, several challenges remain:

1. **Heterogeneity of renal pathology**: Renal lesions in AIDs are various, coming in the form of amyloidosis or glomerulonephritis and vasculitis among others, and thus need to be treated individually [4].
2. **Drug toxicity**: Most immunosuppressants and NSAIDs bear nephrotoxic potential particularly when utilized in the long-run or as combinations [18].
3. **Genetic variability**: In most cases, the reaction to colchicine or biologics (e.g., IL-1 blockade) is also determined by the mutations of certain genes (e.g., MEFV in FMF) [19].
4. **Pediatric population**: The risk level of developing kidney damage with the intake of drugs is higher in children, and they require smaller doses and closer monitoring [20].
5. **Lack of robust clinical trials**: Although registry data (e.g., AIDA Network) are available, randomized controlled trials (RCTs) remain limited and this is more so in patients with comorbid CKD [21–23].

• Moving forward

- **Personalized therapy** It is vital to guide through genotype–phenotype correlation and predict the drug pharmacokinetics [19].
- **Regular renal monitoring**, dose adjustments based on renal function (Table 2), and interdisciplinary management (rheumatology, nephrology, genetics) are critical.
- Future directions should prioritize **biomarker discovery**, **early renal screening**, and **longitudinal data** on newer agents in AID-specific registries [21–23].

**Table 2:** Therapeutic recommendations in monogenic AIDs with renal involvement

Drug/class	Indicated AIDs	Mechanism/pathway targeted	Renal function adjustment	Comments/notes
Colchicine [20, 21]	FMF, TRAPS (off-label)	Microtubule inhibition → ↓ SAA & inflammation	CrCl 30–50: usual dose CrCl < 30: 0.3 mg BID with monitoring	First-line in FMF, effective in amyloidosis prevention
Anakinra [22]	FMF (resistant), CAPS, TRAPS, MKD	IL-1 receptor antagonist	CrCl 30–50: usual dose CrCl < 30: 100 mg on alternate days	Rapid action; monitor for injection site reactions
Canakinumab [19, 22, 31]	FMF (resistant), CAPS, TRAPS, MKD	Anti–IL-1β monoclonal antibody	No dose adjustment across CKD stages	Preferred IL-1 blocker in renal impairment
Etanercept [21–23]	DADA2, TRAPS	Soluble TNF receptor	No renal dose adjustment	Useful in vasculitis-associated AIDs
Infliximab [21–24]	TRAPS	Anti–TNFα monoclonal antibody	Use with caution; monitor renal function	Alternative if etanercept fails
Adalimumab [21–23]	TRAPS	Anti–TNFα monoclonal antibody	No renal dose adjustment	Fully human monoclonal; similar to etanercept
Tocilizumab [17, 25]	AIDs with ↑ IL-6	IL-6 receptor blockade	CrCl ≥ 30: no adjustmentCrCl < 30: not well studied	Consider if IL-1 blockade fails, especially in amyloidosis
Tofacitinib [25]	SAVI, CANDLE (interferonopathies)	JAK1/3 inhibition → ↓ IFN signaling	CrCl < 30 or dialysis: max 5 mg/day, dose post-dialysis	Avoid in ESRD; useful in type I interferon-driven AIDs
Baricitinib [25]	SAVI, CANDLE	JAK1/2 inhibition	Avoid in severe CKD and dialysis	Effective in vasculopathy, monitor blood counts

*FMF* Familial Mediterranean Fever, *CAPS* Cryopyrin-Associated Periodic Syndromes, *TRAPS* TNF Receptor–Associated Periodic Syndrome, *MKD* Mevalonate Kinase Deficiency, *DADA2* Deficiency of Adenosine Deaminase 2, *SAVI* STING-Associated Vasculopathy, *CANDLE* Chronic Atypical Neutrophilic Dermatosis with Lipodystrophy and Elevated temperature, *BID* twice per day, *CrCl* creatinine clearance, *IV* intravenous, *JAK* janus kinase, *PO* per oral, *TNF* tumor necrosis factor

The use of IL-1 inhibitors in AIDs has been included in published European expert consensus recommendations and in Turkish CAPS, TRAPS, and FMF expert consensus statements and has also been reported in the International AIDA Network Registry [16, 24]. The evaluation included regulatory approval by health authorities, and access as per the national drug reimbursement criteria was also considered. For this review, the latest ongoing clinical trials of specific drugs in inflammasomopathies were considered. Overall, 14 emerging potential novel therapies were in ongoing trials based on their listed status. These drugs represented several emerging targeted therapies, including: XENP 313, CER-001, durlobactam-dapson, Dapson EC, Derazantinib, Linzagolix, LNP023, Mavacamten, MW11, R571, Vilobelimab, Tadekinig Alfa, LENABMID1/NL-201 [12–15, 25–27].

Anakinra and canakinumab are IL-1 inhibitors that act on the IL‐1 process that plays central role in inflammation. The fact is that IL-1 inhibitors prevent the inflammation process and contribute to the decrease of SAA levels. This decrease in inflammation and SAA levels reduces the development of amyloid material and the ability of amyloid to injure kidneys [24, 28]. Canakinumab can be considered a first-choice treatment option for patients with renal impairment due to its pharmacokinetic properties being largely renal-independent [28] (Table 3). Here are the key points supporting this:

1. **Renal-independence**: Canakinumab is primarily eliminated through catabolism rather than renal excretion. This means that its clearance is not significantly affected by renal function, making it suitable for patients with varying degrees of renal impairment [24, 28, 29].
2. **Consistent pharmacokinetics**: Studies have shown that the pharmacokinetics of Canakinumab remain stable and do not exhibit significant changes when administered to patients with renal issues. This stability is crucial for ensuring effective and predictable dosing in this patient population [24, 28, 29].
3. **Safety profile**: The lack of renal dependence in its pharmacokinetics suggests that Canakinumab may have a favorable safety profile in patients with renal impairment, as it is less likely to accumulate to toxic levels compared to drugs that are primarily renally cleared [24, 28, 29]

**Table 3:** Canakinumab and other biologics: renal pharmacokinetics and registry evidence

Biologic	Renal clearance	Dose adjustment	Evidence source
Canakinumab [19, 22, 31]	No	Not required	AIDA Registry, PK study
Anakinra [22]	Yes	Yes (< 30 mL/min)	FDA label, clinical studies
Etanercept/Adalimumab [21–23]	No	Not required	DADA2 data
Tocilizumab [17, 25]	No	Use caution in ESRD	Case series, amyloidosis cohorts
Tofacitinib [25]	Yes	Limit to 5 mg/day in CKD	IFNopathy trials
Baricitinib [25]	Yes	Avoid in severe CKD	Pediatric vasculitis reports

*DADA2* Deficiency of Adenosine Deaminase

Observations in International AIDA Network Registry and of some national registries (e.g., the Turkish FMF Network and Italian CAPS Registry) indicate positive results in terms of efficacy and safety of Canakinumab in monogenic AIDs with renal manifestations (especially FMF, TRAPS and CAPS) even in established CKD. There were continued decreases in the SAA and stabilization or amelioration of proteinuria in AA amyloidosis by registry cohorts (Table 3). Canakinumab is considered as a first-line agent in colchicine-resistant FMF, especially when renal amyloidosis exists. A significant benefit of its use is the fact that it is eliminated without affecting the kidneys unlike small molecules or drugs that undergo renal clearance [16, 24] (Table 3).

## Drug-induced nephrotoxicity in monogenic AIDs

### Nephrotoxicity mechanisms and pathophysiology

Certain medications can trigger severe kidney disease in up to 60–70% of patients with a genetic risk factor. Drugs can cause direct cellular toxicity and immune system alert in the kidneys. Injury to proximal tubular cells and the glomerular microcirculation are key factors in the pathological process. Drug-induced nephrotoxicity can be initiated by cellular organelle injuries or by an inflammatory reaction. Loss of a few tubular cells leads to the accumulation of proteins and oxidative stress, culminating in cell death. In CKD patients with diffuse atherosclerosis, starting renin–angiotensin–aldosterone system (RAAS) blockers can resolve macroalbuminuria in about 30% of cases. This is due to reduced kidney perfusion. Patients with monogenic autoinflammatory diseases are susceptible to drug-induced kidney damage. Genetic pre-lesions in tubular cells cause inflammation and vasoconstriction in the glomerular microcirculation. Liver dysfunction, hemodynamic instability, and severity of underlying diseases increase the risk of acute drug-related kidney disease. Drugs can also contribute to the development of tubular diseases. RAAS blockers have limited effectiveness in improving kidney function [30–33].

#### Commonly used drugs associated with nephrotoxicity in monogenic AIDs

Commonly used drugs in systemic autoinflammatory diseases with potential nephrotoxic effects include glucocorticoids, NSAIDs, immunosuppressive therapy (e.g., methotrexate, cyclosporin), and biologic agents. Monitoring pediatric patients with monogenic AIDs is crucial due to the nephrotoxic capacity of these medications. Risk/benefit balance should be considered when using these drugs, as individual variability/resistance may affect their nephrotoxicity. Evidence-based decision-making is important to avoid over-diagnosis and extreme clinical decisions [34–36].

#### Clinical presentation and diagnosis of drug-induced nephrotoxicity

The clinical manifestations of drug-induced nephrotoxicity are primarily acute renal injury. Symptoms include decreased urine output, edema, palpable kidneys. Diagnosis is based on clinical context. Symptoms vary, but hematuria, red blood cell casts, increased pyuria, and renal pain may not always be present. Patients’ drug history is crucial. Renal toxicity is diagnosed through imaging and exclusion of other causes. Multidisciplinary management is useful for patients with systemic symptoms or drug-induced responses. Historical and clinical features help guide clinical suspicion. Signature analysis may not always be obtained. Knowing potential nephrotoxicity can prevent further damage [37–41].

#### Prevention and management strategies for drug-induced nephrotoxicity

Physicians and patients on nephrotoxic medications should be cautious when using anti-inflammatory drugs for monogenic autoinflammatory diseases. Renal function should be monitored frequently, and patients should be called in for follow-up if function decreases. Both clinicians and patients should be aware of possible adverse effects when selecting a treatment protocol. Interdisciplinary monitoring and dose adjustments can minimize nephrotoxic effects. Drug administration in the morning may be beneficial. Careful monitoring and information to patients are necessary to balance risks and benefits. Regular follow-up is crucial for early diagnosis and appropriate treatment [42–45].

During the management and follow-up of various AIDs, renal dosage modification for all medications utilized is required (Table 2).

In conclusion, understanding the mechanisms of drug-induced nephrotoxicity, awareness of their potential adverse events, and applying preventive measures are critical to manage monogenic AIDs with renal involvement safely.

## Conclusive remarks

The review stresses the importance of monitoring renal function, adjusting medication dosages, and considering alternative therapies to minimize nephrotoxic effects. It advocates interdisciplinary approaches to patient care, ensuring that treatment plans are communicated effectively and that patients are educated about potential risks. Overall, the review aims to provide a holistic understanding of monogenic AIDs, their renal implications, and the pharmacological strategies that can be employed to manage these complex conditions effectively.

## Data Availability

No datasets were generated or analysed during the current study.

## Abbreviations

**AIDA**: Autoinflammatory diseases alliance
**AIDs**: Autoinflammatory diseases
**AKD**: Amyloid kidney disease
**AKI**: Acute kidney injury
**ARDS**: Adult respiratory distress syndrome
**ATN**: Acute tubular necrosis
**CANDLE**: Chronic atypical neutrophilic dermatosis with lipodystrophy and elevated temperature
**CAPS**: Cryopyrin-associated periodic syndromes
**CKD**: Chronic kidney disease
**CrCl**: Creatinine clearance
**DADA2**: Deficiency of adenosine deaminase 2
**DIC**: Disseminated intravascular coagulation
**DIRA**: Deficiency of IL-1 receptor antagonist
**FMF**: Familial mediterranean fever
**FSGS**: Focal segmental glomerulosclerosis
**GN**: Glomerulonephritis
**IFN**: Interferon
IL: Interleukin
**JAK**: Janus kinase
**MAS**: Macrophage activation syndrome
**MKD**: Mevalonate kinase deficiency
**NAKD**: Nonamyloid kidney diseases
**NSAIDs**: Nonsteroidal anti-inflammatory drugs
**PAN**: Polyarteritis nodosa
**PFAPA**: Periodic fever, aphthous stomatitis, pharyngitis, and adenitis syndrome
**RAAS**: Renin-angiotensin-aldosterone system
**RCTs**: Randomized controlled trials
**RRT**: Renal replacement therapy
**SAA**: Serum amyloid A
**SAVI**: STING-associated vasculopathy
**TNF**: Tumor necrosis factor
**TRAPS**: TNF receptor-associated periodic fever syndrome
